# Against Single Stories of ‘Left Behind’ and ‘Triple Win’: On Agricultural Care Chains and the Permanent Subsistence Crisis

**DOI:** 10.3389/fsoc.2021.590760

**Published:** 2021-05-31

**Authors:** Dina Bolokan

**Affiliations:** Department of Social Sciences, Centre for Gender Studies, University of Basel, Basel, Switzerland

**Keywords:** labor migration, international division of reproductive labor, agricultural care chains, Moldova, well-being, subsistence crisis, decolonization, post-Soviet studies

## Abstract

The single story of Moldova as the “country without parents” is unsettling. While it is true that villages in Moldova, as in other post-Soviet regions and global peripheries, are affected by intensive outmigration and labor mobility, the image is incomplete. In this article, I propose a different telling of this story: one that looks at and challenges the structural power relations visible in people’s lives in rural Moldova. It is a telling that points to the overall subsistence crisis in Europe and the relationship between neocolonial entanglements and agricultural care chains. As such, this article aims to bring together reflections on labor migration, well-being in rural areas and the global care economy while seeking to decolonize subsistence production and the abolition of the international division of (re)productive labor.

## Prolog

**FIGURE 1:**
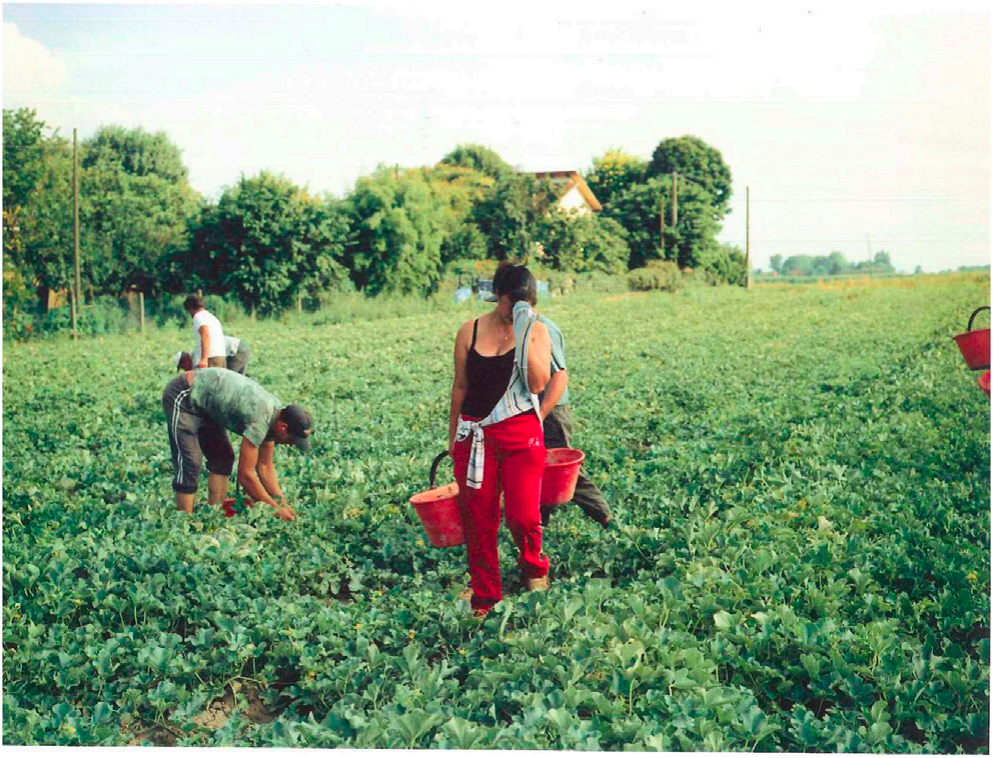
From the series Land ohne Eltern (land without parents). Photo Andrea Diefenbach (2012).

The photo (Figure 1) looks like a field in Italy. It could also be in Germany or Switzerland or England or the Netherlands. A few people are harvesting. Are they all workers from abroad? Are they parents? This is what the book tells us. “A country without parents” is a photo series of Moldovan workers in Italy. It could be a story about illegalized agricultural workers, as Moldova is not part of the European Union so people are not actually allowed to work in Italy, Germany or anywhere else in the European Union. Or it could be about how people from Moldova can obtain Romanian citizenship and work legally if they prove that their ancestors lived on the territory of great Romania before the soviets colonized Moldova. Most people would not call this colonization, but I do, and some others in Moldova do too. Newspapers in wealthy countries like to talk about these poor regions in Europe. There is a discourse in society about people leaving children with their grandparents in their home countries. They call it the ‘left behind children phenomena’. In fact, many people in western Europe like to talk about the ‘poor’. They also like to help people in poor countries, especially when it involves children. It makes them feel better to do so or to buy organic food. But these people find it offensive if somebody talks about how they profit from those ‘poor’ people. How those people are ‘poor’ so ‘we’ can be rich. This is not to be questioned, and responsibility is left to politicians, if to anyone at all.

Back to the photo…

One woman is wiping away her sweat. Is it because it’s hot and the sweat is dripping down and stinging her eyes? Maybe it’s not the sweat she is trying to wipe away but her invasive thoughts. Is her fatigue because her child just called to say that grandmother is sick and she should come home to take care of both of them? Is it because she is physically tired? Tired of the back and forth from Moldova to Italy every couple of months? Or tired because she cannot go back and see her loved ones because of her legal status? Is it because she realizes that after working the whole summer on the field she is still not able to send back money, as living in Italy is expensive, and she has started to ask herself what she is still doing here, and if going back is better or worse. Or maybe it’s because the caporale takes away all the money she earns so she has nothing left after paying for accommodation and food. Is it because she heard about the protests in the nearby village of Rosarno after the racist attacks on workers? Is it because she is scared of what this could mean for her? Of how she, who is not Italian but could be read as such, could somehow be affected by all of this?

So maybe she *is* wiping away her thoughts. Maybe she is not sure how to deal with it. She has her own issues with people who receive more money than she does. *Am I also racist?* she might be thinking to herself. She feels sorry for them but does not protest when others make strange comments about the racist attacks. She does want to support the uprizing workers, but would she then lose her job?

Lost again.

Maybe her feebleness today is because a caporale offered her a deal for sex instead of paying him to bring her to the field and back every day. Or is it because her husband got drunk last night, saying he cannot stand the work anymore, that he’s sick of still not earning enough and not being a ‘proper’ husband, a ‘real man’ who is capable of feeding the family? Is it because he hit her and excused himself at the same moment, promising not to do it again? Is she wondering if this was really the last time, because it was certainly not the first. Probably it’s because she had a great evening last night, laughing and dancing the whole night and now she is tired as she begins her 12-h workday in the field. Or is it because she regrets asking to work in the hot field with the men instead of in the packing hall with the other women, hoping that the change of working position and rhythm would take away her pain? Or does she feel the weight of this all at once? She feels like she is carrying the whole world on her shoulders and life is a never-ending drama with no alternatives in sight. That’s why she has been doing this damn job for years while dreaming of working in her village’s kindergarten again or of finally bringing her children and parents to settle down in Italy.

Why not challenging assumptions? Why not following multiple complexities?

## Introduction

*“Once in a village that is burningbecause a village is always somewhere burning.And if you do not look because it is not your village it is still your village (…)”*

— *Elana Bell (in “Your Village”, Eyes, Stones 2012)*.

**FIGURE 2:**
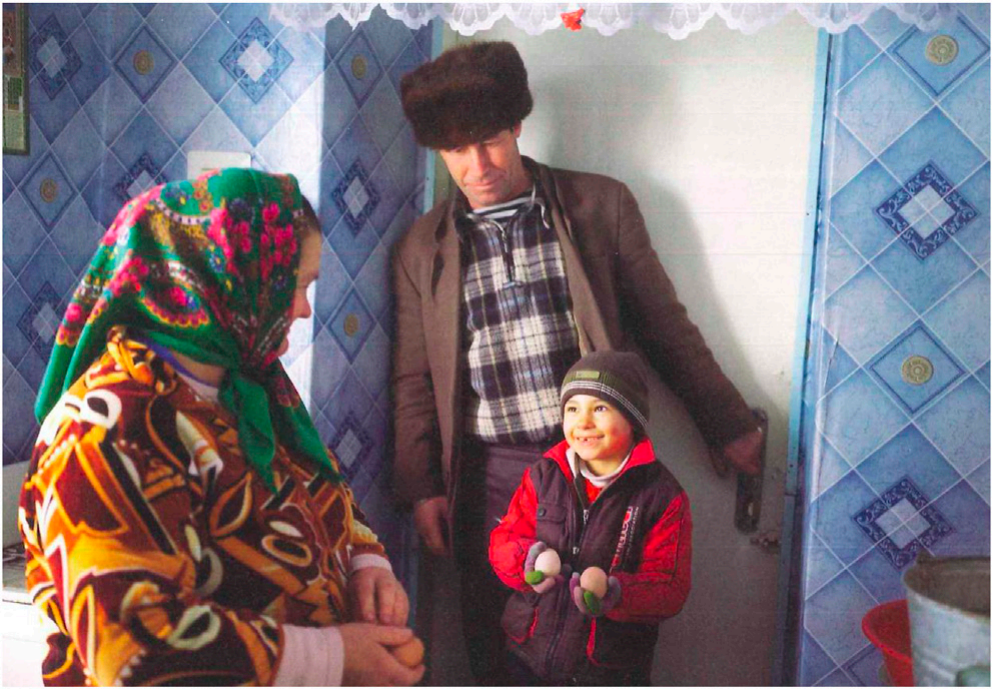
From the series Land ohne Eltern (land without parents). Photo Andrea Diefenbach (2012).

**FIGURE 3:**
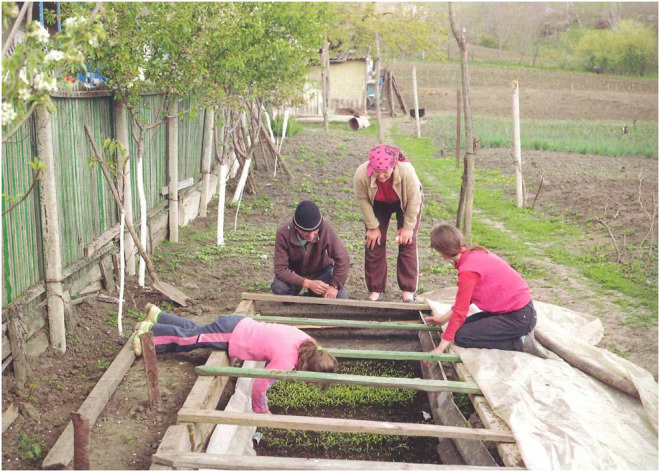
From the series Land ohne Eltern (land without parents). Photo Andrea Diefenbach (2012).

This article looks at the experiences of people from rural Moldova^1^ aiming to reflect on the current subsistence crisis. Villages that are affected by outmigration are thereby understood as crucial starting points of reflection to counter various single stories within this context. “From the Black Sea to the Adriatic, the issue of falling population numbers is a drama. In Moldova, it is a trauma” (Judah, 2020). Indeed, what Tim Judah is pointing out is visible in the depopulated villages of rural Moldova and in other rural regions in post-Soviet countries. Together with Ukraine, Moldova is ranked as the poorest country in Europe, a label that many are familiar with. Within this context, many people joined the global economy, leading to a mass exodus. Moldova is indeed badly affected: today's population is a third of what it was thirty years ago. This reality is taken up in media and documentaries as a one-sided story where Moldova is referred to as the “land without parents” (Flamminio, 2011; Diefenbach, 2012, see Figures 2, 3) or the ‘left-behind children country’ (BBC, 2011; Financial Times, 2015).

Chimamanda Adichie has pointed to the danger of a single story as it “creates stereotypes, and the problem with stereotypes is not that they are untrue, but that they are incomplete. They make one story become the only story.” According to Adichie, this is where power is situated, it “is the ability not just to tell the story of another person, but to make it the definitive story of that person.” The danger lies in the consequences of such a single story as it “robs people of dignity. It makes our recognition of our equal humanity difficult. It emphasizes how we are different rather than how we are similar” (Adichie, 2009). This one specific narrative around Moldova carries all this and a further consequence: it compromises already marginalized communities while discursively placing the blame on those who are structurally most affected by the current care crisis—that are at the end of care chains. The single story of the ‘left behind children country’ does not look into structural power relations and thereby invisibilizes uneven access to and distribution of care capacities. The danger lies in the fact that it contributes to the acceptance of policies that further push for the maintenance of what Spivak refers to as sustainable underdevelopment (2012) and power relations based on colonial continuities.

Nicoleta Esinencu was one of the first authors in Moldova to publicly challenge structural power relations from within Moldova, by destroying the one-dimensional image and perspectives of ‘the good life’ in Europe, thereby challenging another single story. Through her theater play “FUCK YOU, Eu.Ro.Pa!” Esinencu (2005) criticizes the European Union for the economic gap between the poor and rich. This was a direct frontal attack that was not welcomed by the authorities in Moldova or Romania and led to partial censorship. Esinencu also deconstructed the images circulating in western media that portray people as victims. At the same time, she spoke to her fellow citizens, criticizing that “people passively accepted communism, and then passively accepted capitalism” (di Mauro, 2014, 4). Di Mauro argues that in doing so Esinencu “tries to give voice not only to Moldovans ‘lost’ in a difficult transition since 1989, but also to all those societies which do not recognize themselves in the mechanisms of capitalism.” (ibid. 6).

While it has been stated that Moldova is facing a mass outmigration, these labor movements are better characterized by hypermobility in which people regularly commute between Moldova and a place abroad to perform wage labor (Bolokan, 2021). This mobility regime has been implemented into a ‘circular migration’ policy that is referred to as a win-win-win solution. This development strategy is the basis for bilateral agreements between the European Union’s member states and countries outside the European Union that aim to recruit workers for a limited amount of time, thereby revealing the colonial patterns incorporated in post-Soviet and neocolonial labor regimes (Bolokan, 2020). One of the first agreements was with Moldova, where this type of recruitment has become institutionalized and where non-/governmental organizations and further stakeholders control and manage these transnational flows. In the circular migration narrative, it is said that everybody wins: the wealthier countries facing labor shortages, the poorer countries facing a precarious economy and the affected communities, as they no longer have to face brain and care drains since relatives working abroad come back regularly. By looking into testimonies of people living in Moldova that have relatives working in the European Union, I propose to add layers also to this single story represented by the EU’s ‘triple win’ narrative as a solution to the ‘land without parents’ problem.

As this special issue seeks to think about well-being in rural areas in the context of labor migration, I argue that this question can be addressed best if we take “the lives and interests of marginalized communities” as starting points of reflection (Mohanty, 2003, 231), access structural understandings and reevaluate the use of care as a concept. I nurture this understanding from the implications of the new international division of (re)productive labor and struggles within the global care economy. I bring rural care into this discussion to focus on a blind spot in current reflections. Many studies have pointed out that the global care economy and transnational labor migration are accompanied by care chains (Ehrenreich and Hochschild, 2002; Parreñas, 2005; Lutz and Palenga-Möllenbeck, 2012, among others). Care, from a rural subsistence class perspective comprises of caring for and with humans, animals, plants and the soil. For most agricultural workers and for most people worldwide, care is not limited to humans only. Therefore, the care concept, which has focused on human to human relations only, carries an inherent exclusive urban class perspective. I argue that dominant understandings of what care encompasses have therefore been insufficient to grasp the realities of migrating (agricultural) workers and their communities from rural regions. Broadening the understanding of care and care chains can hence shed new light on the current care crisis that becomes manifold in the *subsistence crisis*.

To build this argument, I first trace back the discussions around care from a global perspective and then argue for the need to situate rural caring relations. I then reflect on my methodological and theoretical framework of mapping *rural topographies of care* to present testimonies of people in rural Moldova that live out of agricultural subsistence production and have family members working abroad. In doing so, I diversify the picture of the single story of the ‘left behind’ as well as the single story of the EU’s ‘triple win’ solution by mapping personal coping strategies and structural challenges in post-Soviet Moldova. This analysis reveals *landscapes of wounds* within the context of an overall subsistence crisis that is embedded within global power relations. In the end, I propose a discussion on healing within a transnational perspective on abolition.

## Literature Review: Care Work, the Global Care Economy and Care Chains

In the last two decades, the term care has emerged as an important analytical tool in many social science fields. This can be seen as a product of various feminist interventions, such as the institutionalization of women and gender studies and struggles around unpaid housework in the 1970s. These interventions became known on an international level as the ‘housework debate’ (Hausarbeitdebatte) with campaigns on wages for housework (see Wages for Housework Committees, Lotta Feminista). In this context, committees such as one in London, called for a broader perspective: “Women do the work of producing and reproducing the entire workforce at home, on the land and in the community, in churches, schools and community groups, through voluntary labor and unwaged subsistence farming […] We are a network of Black/Third World women claiming reparations for all our unwaged work including slavery, imperialism and neo-colonialism.” In their understanding, care work encompassed unwaged subsistence farming, health care, voluntary labor and hence reached far beyond the understanding of housework. These marginalized interventions on wages for housework that fundamentally connected to unwaged work in the context of slavery and neocolonialism aimed for an inclusive perspective on liberation in order to “free the whole planet and win liberation for all working people, waged and unwaged” (The International Wages for Housework Campaign, 2020). In this context, the concept of self-care has become influential. It has been discussed broadly since the 80s in Black and queer feminist circles as a tool for survival. For Audre Lorde, this means: “Caring for myself is not self-indulgence, it is self-preservation, and that is an act of political warfare.” (Lorde, 1988, 205). So, when a person is marginalized in society, self-care is an act of resistance. This understanding stands in profound opposition to today’s dominant understandings of self-care (read: self-optimization) in the context of the neoliberal post-welfare states. Radical self-care hence became a crucial point of reference for organizing (for current examples see The Icarus Project, GirlTrek health movement and Radical/Queer/BIPoC Herbalism Networks, among others and see Ahmed, 2014; Richardson, 2018; Hobart and Kneese, 2020).

The so-called integration of women into the labor market did co-occur with the growth of the global care economy (Yeates, 2004). And the gendered division of labor developed along with the nuclear family and the development of the bourgeoisie (Maihofer, 1995), resulting in housework being put on women and wage labor being put on men. This slowly became intertwined with gendered labor regimes, where increasingly women from poorer regions worked in the households of wealthier families so that wealthier women could perform wage labor. The migrating women would then often delegate their care responsibilities, paid or unpaid, to other women in their countries of origin, leading to a redistribution of inequalities that has been problematized: “At both ends of the migratory stream, they have not been able to negotiate directly with male counterparts for a fairer division of household work but instead have had to rely on their race and/or class privilege by participating in the transnational transfer of gender constraints to less-privileged women.” (Parreñas, 2000, 577). The international division of reproductive labor (Parreñas, 2000) and the global care economy are therefore accompanied by global care chains (Ehrenreich and Hochschild, 2002) leading to nanny chains (Hochschild, 2000) and care drains (Parreñas, 2005; Lutz and Palenga-Möllenbeck, 2012) and going hand in hand with ethnicized labor relations (Parreñas, 2001) and care extraction (Wichterich, 2019). Contributions have argued that care capacities are accumulated in the so-called Global North and lacking in the Global South (Ehrenreich and Hochschild, 2002). The new division of domestic labor (Lutz, 2011) has also been understood as something that carries historical continuities of exploitation. Therefore, “colonial ties are often significant in understanding why GCC [global care chains] have emerged and the form in which they developed.” (Yeates, 2012, 141).

Further research reflected on transnational (nuclear) families, focusing on the challenges and opportunities of transnational motherhood and fatherhood and the effects on children (Hondagneu-Sotelo and Avila, 1997; Bryceson and Vuorela, 2002; Dreby, 2007; Mazzucato and Schans, 2011; Ducu, 2018) as well as changing gender relations within transnational care arrangements (Lutz and Palenga-Möllenbeck, 2016) and the possibilities and difficulties of parenting at a distance through mobile phones and new technologies (Madianou and Miller, 2011; Nedelcu and Wyss, 2016). The important contribution of the remaining grandparents was also highlighted, while at the same time pointing to the constituting role of migrating grandmothers in the global care economy and in post-Soviet nation-state building (Solari, 2017). Following the care chain approach, it has been made visible how these chains in Europe reach, for example, from Ukraine to Poland and from Poland to Germany (Lutz and Palenga-Möllenbeck, 2010, 2011). While it has been stated that these chains are always in flux (Murphy, 2014, 192) and have therefore different spatial and temporal characteristics, the concept has so far not been discussed regarding rural to rural/urban labor migration. Suggestions have been made on how to further deepen the analysis of global care chains such as looking at additional occupations and more countries worldwide (Yeates, 2012, 147) and considering the elderly (Escrivá, 2005, 14). But is has also become clear that “it is insufficient simply to gather more empirical data from diverse locations; rather, we need to take on board what these different localities can contribute to questions and expanding our conceptualizations and theorizations.” (Raghuram, 2012, 160).

I conclude that reflections on care, the global care economy and resulting care chains have produced a broadly resonant discussion in the social sciences and society. However, they have also led to a narrow understanding of the care (chains). As a result (I) care has hardly been understood as the production of life in the broadest sense (II) discussions of transnational care relations have lost their emphasis on the historical weight of colonialism and exploitation in a global, patriarchal and neocolonial world and (III) the important notion of care as self-care and as a practice of resistance for marginalized communities got lost. As a consequence, the specific challenges of those workers and their communities that come from rural areas and are involved in subsistence agriculture, was overlooked. I hence propose 1) to reflect on care from an epistemological as broad as possible but socioeconomic embedded and place-based perspective, thereby deconstructing the binary-western-patriarchal-colonial understanding of care and 2) to reevaluate the notion of care chains in the context of migrating agricultural workers from rural Moldova, thereby 3) developing an *intersectional perspective on care*, that also opposes scientific single stories such as disciplinary thinking that narrow theoretical and empirical approaches. I hence open a multidimensional discussion on caring relations that beyond multiple empirical and epistemological perspectives on rural Moldova also allow for further theoretical implications on the global care economy, the crises of (re)production and on gender theory.

## Situating Care (Chains) as Agricultural Care (Chains)

This article is concerned with what happens at the end of the care chains in Moldova, as the majority of the rural working population undertakes wage labor abroad. I already elaborated on what *hypermobility* means for those that leave regularly to work in Europe's agricultural and agrifood sectors (Bolokan, 2021). Here I follow up on these reflections while looking into what *hypermobility* and migration mean for those that remain and take care of subsistence production. What are the challenges and involved global power relations in rural Moldova and how do people cope with being at the end of care chains? In this section I will reflect on the epistemological dimensions of care and the socioeconomic dimension of care work under capitalism in order to situate care chains and rethink care in the context of translocal rural to rural labor mobility and migration.

### Epistemological Approximation

In the social sciences today, care is often referred to as a “social and emotional practice that does not necessarily need to be defined in relation to the spheres of work but rather entails the capacity to make, shape, and be made by social bonds” (Alber and Drotbohn, 2015, 2). Care, as referred to in the social sciences, therefore carries a dimension of interpersonal relationships (relationships to other persons). Therefore care is mostly referred to as caring for (including tasks such as cooking, cleaning, washing, listening and healing) and caring about (working on the relationship between people and the development of their bonds) (Yeates, 2012, 138 referring to Lynch and McLaughlin, 1995, 256–257). I argue that the emergence of care as a concept goes back to the distinction of people in industrialized societies as male breadwinners and female nurturers. These gendered and hierarchical oppositions have not only displaced people from their subsistence (primitive/origin accumulation) and divided human tasks into paid wage labor and unpaid care work, they have also led to a definition of care from a human centered perspective as something that people do to each other. Under this logic, people are either care receivers or care givers. *I argue that the dominant perception of care is fundamentally linked to a western, patriarchal and urban biased understanding of relating to and taking responsibility for the world.* I further argue that this human centered understanding of care stands in the tradition of binary thinking. As a consequence 1) it detaches human animals and their well-being from ‘the rest’ and hierarchizes relations. 2) This hierarchization again builds the condition to devaluate entities that are not human animals and belong to ‘the rest’. This ‘rest’ is put as being closer to ‘nature’, the very logic that racializes people and degrades women, non-binary persons such as all people that are not being identified as the white, abled, heterosexual, Christian, cis man. Historically this logic developed along the justification to exploit ‘nature’ and enslave people, a process that not only did harm to the colonized and their territories but came back to the colonizers and to Europe (see oppression of ‘nature’—internal as well as external—and intrahuman forms of domination and oppression/connection between domination of nature and domination of humans, in Horkheimer and Adorno, 1947). Toni Morrison described it as follows: “(…) They had to dehumanize, not just the slaves but themselves. They had to reconstruct everything in order to make the system appear true.” (Morrison in Gilroy, 1993, 178). This led to inner colonization in Europe (Ha, 2008) and to “‘an arc’ of colonialism-nationalism-fascism” (Aikens et al., 2019, 9). It also went hand in hand with a *devaluation of the rural and subsistence production*, that was seen as the non-modern and backward side of society. At the same time, caring and being responsible for was only understood within the relations of the nuclear family (the caring mother), wage labor relations (the caring patron) and the nation (the caring state for its citizens). I argue, that the single story of Moldova as “the country without parents” where we are left with a single narrative of relations and responsibilities, also carries a binary thinking such as a colonial understanding of care. Hence to deconstruct the concept of care and to rethink caring relations, opens the space for multiple narratives and stories along with nuanced perspectives on care and caring relations in Moldova and beyond.

Indigenous sciences (Snively and Williams, 2016) and Indigenous knowledge systems (Tippins et al., 2010) have been referred to as Indigenous ways of living in nature (Aikenhead and Ogawa, 2007). This implies a reference system where the concept of living cannot be disconnected from ‘nature’. Human animals are a part of this, which contradicts the western dichotomous understanding of nature vs. humans. These understandings thereby oppose further pairs such as nature vs. culture, biology vs. sociology, reproductive vs. productive and so on. Indigenous theorizing hence refers to “a world in which the multiplicity of living beings and objects are addressed as peers in constituting knowledge and world.” (Sundberg, 2013, 42). As the word concept for care does not exist in all ‘non-western’ languages, it is not about applying *the* indigenous understanding of care to the European context (*the* overall understanding does not exist anyway). Rather, I propose that care from a non-western and intersectional perspective cannot be disconnected from ‘nature’. *Accordingly, I understand care first of all as reciprocity and circularity with and between all entities involved in constituting a relation.* In other words, “if care is to *move* a situation, those who care will be also moved by it” (de la Bellacasa, 2012, 206). This includes the idea that care does not only take place between humans but also between non-humans and other entities. It is therefore a complex dynamic that includes, but is not limited to, the articulating and percipience of needs or feelings.

This comes with many challenges: communication in caring relations is embedded in structures of power and domination and changing ontological limits of entities. It depends on who is able to communicate how and to whom and who is able to understand and receive what has been communicated and to interpret or act accordingly. In an epistemological sense, postcolonial thinkers have discussed the limits of understanding and the need to listen (Spivak, 1988), an important intervention that pushes those in more powerful positions to do their work in “learning to unlearn” (see decolonial thinking, Tlostanova and Mignolo, 2012). Further epistemological implications come with insights in interplant communications (for example Song et al., 2010) and ways of caring between species. A sage plant, for example, starts to activate its defense enzymes and genes when a damaged tobacco plant grows next to it. She understands the warning signals from the tobacco plant (Köchlin, 2020). Reading such caring mechanisms is challenging when the discussion returns to farming people and the impact on their agricultural caring relations; above all, it requires longitudinal and deep observation and transgenerational communication.

### Socioeconomic Approximation: The Political Economy of (Re)productive Labor/Care Work

**FIGURE 4:**
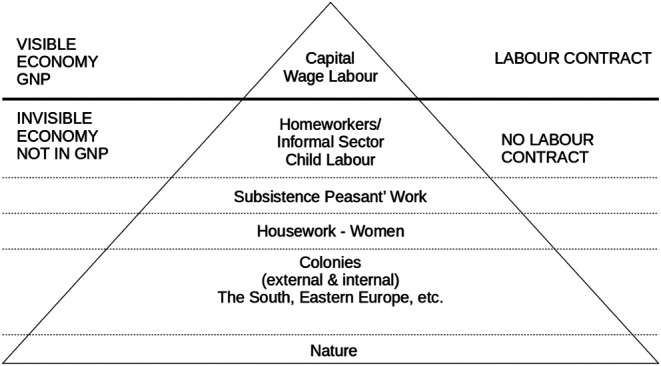
The Iceberg Model of Captitalist Patriarchal Economics (Mies & Bennhold-Thomsen 1999).

Feminists in the 1970s and early 80s deepened reflections on what capitalist and socialist/communist economists have erased in their reconstruction of value production: mainly unpaid, invisibilized and unvalued labor that precedes and goes hand in hand with the production of goods and commodities. Reproductive work could be understood as the work necessary to produce and maintain humans, a kind of human labor that unlike domestic work also includes childbirth, raising children, and emotional care and attention. This division has been criticized, however. Why should only the production of goods and commodities be considered ‘productive’, and why not the ‘production’ of life and the maintenance of living processes? In contrast to Karl Marx^2^ and following Rosa Luxenburg^3^ (Mies and Bennhold-Thomsen, 1999, 30f), the subsistence theorists (also referred to as the Bielefelderinnen or The Bielefeld School) developed the Iceberg Model of Capitalist Patriarchal Economics (Figure 4) in order to map the invisible economy that produces value and is exploited under capitalism; it does so without dividing labor into productive and reproductive forms.

The outlined spheres of the invisible economy represent different areas of the externalization of costs that are treated under capitalism as free goods to be taken and appropriated. This immense volume of work bears the foundation of capitalist surplus-value. Meanwhile, subsistence peasant work, housework and so on are taken together under the term subsistence production:

“Subsistence production encompasses all work that is spent in the production and maintenance of life and also has this purpose. Thus the concept of subsistence production is in contrast to the production of goods and market value. In subsistence production the goal is ‘life’. In the production of goods, the goal is money, which ‘produces’ more and more money, or the accumulation of capital. In this logic life is only kind of a side effect. It is typical of the capitalist industrial system that everything it wants to exploit for free is declared to be nature, a natural resource. This includes the domestic work of women as well as the work of small farmers in the Third World, but also the productivity of nature as a whole." (Mies and Bennhold-Thomsen, 1999).

Following this understanding, the term and concept of subsistence production contains the inseparable connection between domestic and housework as well as the agricultural subsistence that is the human and non-human life production. This includes almost all work beyond wage labor in the colonized and colonizing regions. All of these spheres are considered to be productive labor. Despite the appropriate and important criticism of the Bielefeld School (Attia, 1991; Glenn, 1992, 2; Baier, 2010, 76; Knittler, 2005, 90), I consider the Iceberg Model and the definition of subsistence production to be helpful for further theorizing the political economy of care work and agricultural work as belonging and as the invisible economy of surplus value under capitalism.

In the case of agricultural workers and their communities, caring relations have to be situated within the invisible economy and care has to be understood from a place-based perspective. Many people who work in the European agricultural sector operate as smallholders. While they harvest abroad or work in the food processing industry, other people take care of their social responsibilities toward friends, relatives and neighbors and toward their agricultural subsistence. This affects more than just interpersonal relationships. In the case of agricultural workers and their rural communities, it has an impact on them as smallholders. I, therefore, propose a widening of the perspective on care chains in the context of rural to rural labor mobility and migration by situating rural care chains, using *agricultural care chains* as an analytical framework. This understanding of agricultural work as care and the other way around is meaningful because it carries analogies. These analogies show us the concrete conditions of agricultural work that are defined by cycles of (re)production with their own diverse rhythms and dynamics. Furthermore in small-scale subsistence agriculture, the working spheres are not divided on a functional level but are *spatially interrelated.* Housework, care and agricultural care—that is, living, eating, cooking, feeding people, nurturing animals and looking after plants–take place on the same terrain. Taking this into considerations leads me to look into *rural topographies of care* in Moldova, thereby developing an intersectional perspective on the multiple complexities of rural challenges and perspectives.

## Decolonial Life Course Approach and Rural Topographies of Care

This article focuses on what happens at the end of agricultural care chains thereby adding layers to the single story of “the left behind children country”. I look into the challenges and coping strategies of the elder population^4^ in rural Moldova, as their perspective is crucial to understanding the effects of labor mobility and migration on rural communities and rural spaces. Insights are based on longitudinal research on *hypermobility* in the agricultural sector in Europe that took place over nine years in the context of a *decolonial life course perspective* (Bolokan, 2021) and that weaves together a life course approach with global ethnography from a decolonial and queer feminist perspective. Through research of the effects of globalization on people’s everyday lives, this approach asks to 1) situate people’s life courses within the shared experiences of their communities and to identify the entangled local and global power relations that reveal continuities of colonial exploitation (in this case including the afterlives of Soviet rule), while 2) at the same time (re)searching for perspectives to decolonize oneself and the world.

The *decolonial life course approach* was enriched by personal experiences. I was born in Moldova but migrated to Germany in 1991. Many of my relatives in Moldova live on subsistence agriculture. Growing up I became sensitized toward understanding the overall changes in Moldova after the collapse of the Soviet Union. I regularly go to Moldova to fulfill my care responsibilities toward my relatives. While going back and forth, I periodically meet people that work abroad. At the same time, following their biographies and those of their extended social networks developed into a life course perspective, that was not a disconnected and alienated research strategy, but as a supportive part of my own back and forth movements. Ultimately, it was also a personal coping strategy to meet my own translocal obligations and the demands that accompanied it, meanwhile my interactions with the extended communities had to go far beyond my wage labor in academia. So from the beginning searching for emancipatory perspectives on well-being, become and remain a constitutive part of my knowledge production.

Within this context, the vignettes below stem from biographical interviews and conversations with 22 smallholders that have family members that are temporary or permanently working in the European Union mainly in the agriculture sector or/and the care economy. I was able to meet these subsistence farmers through their relatives that work abroad and that I previously conducted interviews with (34). Others I came into contact with through the social networks of my own relatives and friends in Moldova. I met some of them several times over this long period. At times our contact would include living at their places or close by for a certain amount of time. Most of these conversations took part in rural Moldova and were multilingual, mainly in Russian and Romanian/Moldovan. The length of the talks and interviews ranged from 60 min to 3 h.

Because research on care chains has overlooked rural specificities, I chose to focus primarily on agricultural care. In some cases, longer interview passages will be integrated into the vignettes to give as much space as possible for people to tell their own histories and develop their own narratives–with as few omissions as possible–and to allow a broader context for interpretation than the one that is possible within this framed article. The people and the stories presented are selected in a way as to encompass the experiences, life trajectories and main topics of all 22 smallholders and show how challenges and coping strategies in rural Moldova cannot be disconnected from its post-Soviet past nor from its neocolonial present. This enables a mapping of *rural topographies of care* that are situated within post-Soviet entangled spaces and translocal labor mobility regimes. This mapping is a firs step toward opposing the single story. Using topographies as a tool allows a detailed description of multiple social dynamics as well as a description of rural Moldova, rural areas and agriculture. Following Cindi Katz, “Topographies allow us to look, not only at particular processes in place, but at the effects of their encounters with sedimented social relations of production and reproduction there. In other words, topographies are thoroughly material. They encompass the processes that produce landscapes as much as they do the landscapes themselves, making clear the social nature of nature and the material grounds of social life.” (Katz, 2001, 720)

Thus, topography offers a method for examining the material effects of globalization that are found in social practices as well as inscribed in places themselves. Following Katz, this can be understood as a basis for countertopographies (ibid. 721ff).

“One can imagine mapping places connected along a multitude of different contour lines, each marking a potential terrain of translocal politics. In other words, the political, theoretical, and methodological project I want to advance is one that constructs countertopographies linking different places analytically in order to both develop the contours of common struggles and imagine a different kind of practical response to problems confronting them.” (ibid. 722ff).

In this understanding, identifying such contour lines and the involved translocal politics allow for mapping geographical imagination and countertopographies that are grounded in “multiple situated knowledges” (ibid. 723ff). It hence connects a variety of places affected by similar social phenomena and structural power relations while bringing together a variety of situated experiences and of struggle in order to deepen resistance practices. My methodological proposition here is to follow agricultural care chains and to describe rural topographies of care where people find themselves at the end of these chains and to look for such contour lines.

## At the End of Agricultural Care Chains: Challenges and Coping Strategies

I have already reflected on the inner European recruitment chains of workers toward Eastern Europe within the agricultural and agrifood sectors. They generally reach from wealthier European countries such as Germany, Switzerland, Sweden, England and Denmark to Poland and Romania. Within this context, Poland is one of the most important so called sending *and* receiving countries. As a result, the recruitment of workers from Poland to practically all wealthier European countries comes along with *recruitment chains* that involve workers from Ukraine and Moldova in the context of *post-Soviet and neocolonial agricultural labor regimes* (Bolokan, 2020). Hence Moldova finds itself at the end of recruitment chains within the broader labor market. What the ‘left behind children country’ discourse is hinting toward is this precarious situation of a mass exodus, especially in rural Moldova, thereby producing a single story. However, in reality, these agricultural care chains end due to mass outmigration and hypermobility within whole communities, so different coping strategies arise to maintain subsistence production. In Moldova, around 70% of the population lives from agriculture (Mocreac, 2018, 7), mainly as subsistence farmers. The following life stories allow insight into people’s thoughts, decisions and struggles from a biographical perspective. These life stories present counterstories to the narrative of the ‘country without parents’ and undermine the one-dimensional single story. The people I interviewed are connected by similar situations, as they all have relatives working abroad, mainly in the agricultural and agrifood sectors. Whereas ten years ago almost every household had somebody working or living abroad also supporting the family, these days, most of the members, especially in the younger generations, have some temporary wage labor experiences or even live and work periodically or permanently abroad. Out of the 22 smallholders I talked to, I focus on four farms and six people, so I choose to look into only a few lives to give deeper insights into trajectories and experiences. These experiences stand for themselves but have strong parallels to the other smallholders I talked to. Therefore they are embedded within shared experiences in communities that are affected by hypermobility and mass outmigration.

In order to better understand the agrarian structure and the social power relations in the villages and to situate the experiences below, it is necessary to discuss the *lider system*. I refer to lider as a system, as nearly every village in Moldova has one or more such liders. These *liders* (Romanian/Moldavian word for leader) form a kind of rural upper class that owns machinery and can therefore work the land and can relate to more powerful social networks that allow them to sell their products. After the Soviet Union ended and in the process of decollectivization, the distribution and privatization of land and infrastructure was organized in this way. All the land that belonged to the kolkhoz and sovkhoz farms was divided to the members as small lots of 0.5–1.2 ha called cota. The challenge that subsistence farmers face is that this amount of land is too big to work manually and too small to start a farm. To work this amount of land, several people or some machinery such as a tractor is needed. As a result, people either sold this cota or rented it to those who had a tractor and some knowledge of how to work the land. Initially liders were the former kolkhoz/sovkhoz directors or other people who managed to benefit from the Perestroika and privatization of the remaining agricultural infrastructure. Later, new liders appeared that, for example, earned their money by working abroad and today represent a new class of rural entrepreneurs. Those that lease their land to liders are getting paid with a defined percentage of the harvest. This means that the subsistence farmers carry the risk of a bad harvest, no matter the reason. In general, liders aim to buy or rent more and more land while building an infrastructure that increasingly relies on credits. Today, most of the liders are heavily indebted and strongly dependent on banks. Due to the composition and the history of the liders, their understanding and implementation of agricultural production is harmful toward ecology and the soil. I now invite you to take the time to learn more and to listen to multiple narratives.

## Rodica

Rodica is a remarkably strong woman in her fifties. She runs a farm close to the capital Chişinău together with her husband, Jevgeni. Their three children work regularly abroad in Poland and Italy in construction and gastronomy. Rodica and her husband not only take care of their farm, but also of her sister Lena two children. Lena left her abusive husband in 2009 and has been working in the agricultural sector in Italy since then. Both children are teenagers and cannot wait to be old enough to also leave for Italy. Rodica has a very heavy workload. She works from 5 am until 10 pm. Everyday. She gets up in the morning, feeds the animals, milks the two cows and then travels 45 min to Chişinău in order to bring a few liters of milk to a woman to sell it on the local market. Returning already by 9 am, she prepares breakfast for the children and Jevgeni, who is just then coming home after his night shift at a carpark in the capital. He then goes to sleep; she brings the cows to a field close by so they can graze. After that, she works in the garden and prepares lunch. Sometimes she is lazy, she told me, and goes to sleep for a half-hour after lunch. In the afternoon, they work together on whatever needs to be done. They do not rent their land to a lider. The children used to help but they now work abroad and when they come home after a few months working abroad, they are exhausted and require a proper rest. Rodica wants the children to save the money they earn so they can build their own houses. Her way of coping with the immense workload—that is, taking care of the animals and plants and from two to five children at the same time—is to completely overwork herself every day. She does not accept any help from the children, instead encouraging them to save their energy, recover and focus on building their own futures. She hopes this will not be abroad but close to her. Taking care of herself is an everyday struggle and is only sometimes reflected in a half-hour midday nap.

## Alesja

Alesja grew up in a Ukrainian village in Moldova in a kolkhoz family. As a child, she helped her parents harvest tobacco. Both of her parents, as well as many people in the village, died from the effects of tobacco growing. As a single parent, she tried to create a better livelihood for her children than the one she had herself. She decided to plant fruit trees. Her children, who used to support her in her work, currently all live and work abroad. One daughter lives in Ukraine, the other works in Italy, and her son is in Spain. Alesja has been struggling with this situation for years. She now lives half of her time in Chişinău and half of it close to her orchards, which are two hours away from the capital.

Me: How did you come to run the orchard?

**Alesja:** We have worked with tobacco since we were children. It was strictly forbidden then. But the parents didn't always manage to cope with the work, so we helped. Then when examiners came, the parents hid us. The parents were threatened with punishment if they saw even one child working with tobacco. I started to understand early on why this work was so unhealthy. I then asked myself how I could work with the earth and earn enough to eat and live without having to plant tobacco. I sat down and thought and counted and counted. Trees can give more harvest than tobacco. That also means more income. Working with trees is also much more interesting and beautiful.

Me: Did your parents work on a kolkhoz farm?

**Alesja:** Yes, that was on the kolkhoz farm. It was a catastrophe. It was such unpleasant work. Until now I have memories of it. It was not free will to do this work. We were taken out of bed early in the morning with great effort, at 4 already, because the tobacco had to be collected before sunrise, so that it wouldn't stick together, so that there wouldn't be any dew on it. By 7 the tobacco had to be harvested. Then the car was loaded and the tobacco was driven away. Then it came into a shelter and we put every single leaf on a string with a needle. I can't remember much joy as I have now with the fruits. This work was hard and unpleasant. The hands were black and dirty from this tobacco. Getting the hands clean was a real problem. It took a lot of effort to clean them. You had to use a piece of paper to take a piece of bread and a tomato and eat them. That's how we ate lunch. There were no gloves then. Nobody thought to protect themselves at that time. And the next morning you had to get up and harvest tobacco again. It was hard.

Alesja later moved to Chişinău, had two children and became a single mother after a divorce. When Perestroika began, life was very hard. As the child of a kolkhoz farm family, she did not count as a worker. Still, she fought for her right to receive land and was able to plant trees on it. Until the harvest came, she had to find a way to survive. She went around villages and asked people if she could take fruit from their trees. She gathered jars from everywhere in the city, with the help of the children. Others would have been embarrassed, but she did this always with her head held high. “In those days of Perestroika you just had to see how to get through,” she said. As many others, she sold her preserves at the market. She could not survive in the city because of the high cost of rent and gas, so she moved to the countryside, and was glad to be near her infirm parents. There she grew vegetables between the planted trees in order to feed herself and her children. Her subsistence began to flourish in the place where the tobacco had been. The children helped a lot, especially her daughter, and she was able to start to love agriculture again.

**Alesja:** It’s the children which taught me to love the earth. What love for the earth should I have developed by working with tobacco? But the children were always happy to see such a small seed now become a big plant.

Me : How is it to work in the orchard since the children went abroad?

**Alesja:** The difference is incredibly big. When I knew that my children were in the city, I got up in the morning with such enthusiasm. Early in the morning at 5 I went weeding, collecting wood for the next season. I had so much energy, so much strength, so much will. But now that the children are gone—the worst thing is that they don't have the right documents to come and visit me. When they leave, they can't come back. It's boring without them. I miss them. I no longer have that strength, I no longer have that will. On top of that there are other problems I have now (…) Last year they offered that I sell our orchard. The younger daughter told me to do it. I was in a terrible state. I was alone, the children were gone. No one can help. Alone I'm overwhelmed (…) It wasn't all that easy. And I felt the need to sell. The son also told me to sell, they won't let you work there in peace anyway, he told. They want the land, you have to understand that. They will harm you, they will do mischief there. You can't fight them, they have power, money and who are you next to them. So there was a moment when I wanted to sell, but my daughter Anastasia, who raised the orchard with me, said: The garden is not for sale. I might come back, who knows, maybe I will spend my old days there. The garden means more to her than to me.

Me: She also has worked a lot in the orchards!

**Alesja:** Yes. Every single tree she has grown and with her 24 years she knows that she has her own orchard. After all there are 200 trees there. That is her property (…)

Me: And your son?

**Alesja:** He is in Italy. They have thought and decided that it is best for them at this time. I would like to live with the children, but they are there now. That would be better, they say. But in the conversations with my son I hear that nothing is better there. He's drawn home. Everything is strange there. There is only work, house, work, house and no friends (…)

Me: Many tell me that it is difficult to sell the harvest. How about you?

**Alesja:** I have been thinking for a long time about how to do it and I have come to the conclusion that it is important to process the products before selling them. Here lies the profit (…) If it is of high quality, people will always buy from you. Many have understood what kind of work it is. I put the glasses in the garden, on the fire with the branches of the same fruit trees. I am lucky to meet people who appreciate that (…) These liders who also had peaches did not have as good peaches as I did. They chemically processed their trees. I had to sell it within a week. The transport costs were very high. As soon as the harvest was ripe I only had to call and people came because they knew I had a good product. They paid three or four times more. Everyone said sell it. Export, export. But why sell to people far away when I don't even know who is going to eat them? I'd rather sell to the people around me. You know, the garden has changed me a lot. Every flaw in my life I have jetted with the weeds. A sin here, that doesn't want to come out but it will soon or later (…)

Pressure from the outside to sell the orchard continues to increase over time. The regular harassment and the unclear future prospects of her children contributed to a fundamental lack of motivation. But Alesja keeps trying to maintain the garden on her own while her three children are abroad. She seeks to create a place for them, a space that opens the possibility to imagine a future where they come back and live with her again. She saved money for decades and finally bought her own first car. This new resource gave her the opportunity to bring her fruits by herself to the market and home to consumers without having to pay expensive transportation costs. It also allowed her to visit the elderly home more often and regularly bring Oxana, one of its inhabitants, to the orchards. Oxana is 45 years old but was brought to the elderly home because she is blind and her family was unable to cope. During the harvest time, Oxana helps Alesja in the garden, pitting cherries and peaches. Alesja also tries to involve the village children in picking fruits, giving them a few buckets of the picked fruits in return. She thinks it is also important to teach them how to cultivate and can fruit on the fire with branches from the fruit trees; this enables an ecologically closed circuit and a favorable processing method. She developed this process of reciprocity and circular out of necessity over her lifetime, as she was poor and without infrastructure; it gives her motivation to see nowadays other people reflecting on ecological farming, as she has been doing this for decades already but without this concept in mind. However, after a while the parents did not let the children help anymore. And, her car was burned while she was inside the house. Alesja knows the men who did this and that it happened because she is not willing to sell her orchards, the orchards that mean everything to her, to her daughter and to Oxana.

Alesja has developed many ways of dealing with (seemingly hopeless) personal and political crises over her life course. After working with tobacco plants as a child, she had to develop a new relationship with plants to care for and harvest them. This renewed relationship allowed her to survive but also brought joy and beauty. Her children helped her by sharing their enthusiasm and her relationship with agriculture grew to be reciprocal—the plants helped to solve her problems. If, as she says, she made mistakes, they were put into perspective in her communication with the weeds. However, since the children left, she finds it difficult to maintain a relationship with the work with the trees; now, her motivation fades. She has tried many things to avoid losing herself in loneliness. The mutual relation between Oxana and Alesja was enriching. Sometimes she would also pay people from the village in the intensive harvest weeks, as her children could not help. But paying others turned out to be difficult socially—there can be a lot of envy involved, even anger, she explained. While many years of intensive care and maintenance of the trees finally allow for good harvest, it is unclear whether she will sell her land as she now faces threats and attacks. Of course she doesn’t want to sell the orchards as they are not only a space to escape the city and the elderly home. Still, this place of self-care that nurtures Aljesa’s and Oxana’s spirits, that gives motivation and hope, is in danger recently.

## Ina and Sergej

Ina and Sergej are both in their 70s and live in north Transnistria, close to the Ukrainian border and far away from the capital. This area is especially facing depopulated villages. They have taken care of their farm as well as their two grandchildren for the past six years while their children work abroad. While they were working undocumented for a few years, the children were not able to come back to Transnistria/Moldova. They legalized their status, but the working conditions are so time and energy shaping that they do not allow for childcare and the employer does not allow children on the farm. It was always unclear if they would be able to stabilize their living conditions, so they would plead their parents not to sell the land; although they hoped they wouldn’t be forced to return, it was a possibility. Since the children left, Ina and Sergej have reduced their subsistence to the minimum. They take care of their garden and their two cows, but they cannot work their land anymore and therefore rent it to a lider. They had to do this as they do not trust those around them to organize such an action as buying a tractor together and using it all together. Often when Ina and I meet we reflected on how trustful relations are missing but would be so important. And we reflected upon the lack of a younger generation, that is urgently needed to work on this. Some years later they were not even able to care for both cows anymore: the neighbor who used to bring all the cows in the village to graze in exchange for food is not doing so anymore. In her 70s, Ina earns some extra money by taking care of empty houses, as the owners live in the capital or abroad. Ina is happy to have this opportunity as she sees how the other elderly in their village that do not receive any additional money barely live off their small subsistence. They cannot even afford to become sick, as getting ill could be a death sentence under these living conditions–a topic we also often discussed.

I saw Ina and Sergej in 2013 and 2017. They always hoped that their children would come back home or take the kids and maybe also themselves abroad. Only in 2019 were they able to get the kids. Shortly after, Ina had a stroke. They are not able to immigrate and live together with their children and grandchildren, as so-called family unification is not meant for parents. Now Sergej is taking care of Ina alone. And while the children are not able to support them financially, they still did not sell their land, because who knows, they might still want to come back one day.

## Emilian and Vala

Emilian and Vala both come from families that have been farming for generations in the kolkhoz. They are in their 70s and live in a village close to Hînceşti, around one and a half hours away from the capital. Their village is less depopulated than others. The closer you are to Chişinău, the more populated villages are. Still, all three of their children have been working and commuting between different jobs and countries for many years, and for now they spend most of their time abroad. Because of the lack of physical help, they are only growing grapes; they stopped having animals and reduced vegetable cultivation to a few tomato plants. Their farm became a monoculture farm.

Me: So because you worked for the kolkhoz you received land when they closed down?

**Emilian:** Yes, they divided the land and I got something. But the tractors and the storehouses were not divided. So I had to buy a storehouse. Down there in the village, where the shop is now. That was when people started working for themselves. Not for the farm. But where to sell, I don't know. Some were cheated. They didn't get paid for their products. People got tired of working for nothing. They stopped working the land and that was it. And that's it. But I bought another four acres and planted vines on it. There were still some old vines here. There is still a piece there that I have to make new but for that I need money that I don't have.

Me: And since when do you have this farm?

**Emilian:** Since 1997.

Me: The kids were small, right?

**Emilian:** Yes, one of them was already in school and the daughter too and the other son was still small. Back then we still had cows and sheep. We also had vegetables for our own household. The grapes were for sale.

Me: And the children helped?

**Emilian:** Of course they helped.

Me: And how do they help now?

**Emilian:** A little with money. When they come home they help out too. And the daughter, I show you pictures of her. This is my little granddaughter (showing photos). She's in Italy. And this is my daughter. Here we are at the wine exhibition in Hînceşti. There we got an award. The daughter lives in Italy for many years.

Me: And the elder son?

**Emilian:** He is in France.

Me: And the other son.

**Emilian:** The other son is preparing his wedding. He is now in Switzerland.

Me: And they work in agriculture there too?

**Emilian:** Yes, the son also works there in vineyards. And the other one in France in construction. And the daughter is taking care of an elderly woman in Italy. As usual, Moldovan women do that.

Me: Has the way you work changed since the children left?

**Emilian**: The expenses have become much higher. Now I have to pay for every step. Before the children helped, now they help with money, if they can. They already have their own family. I want them to come back. I want them back very much. We even built a house with the children. They helped too. My daughter and wife cooked for the workers. The sons drove their cars to bring the sand and whatever else was needed.

Me: And if you need help, not with the farm but because you are sick or something, who helps?

**Emilian:** Friends or neighbors.

Me: Friends or neighbors. So people look for each other here?

**Emilian:** Yes, of course. There are 500 houses in the village. 90 of them are empty. There used to be about 1,500 people living here. Now there are about 1,300.

Me: I see that there are still many children here.

**Emilian:** Yes, yes, yes. We still have many people here. Not like in other villages. But not everyone knows how to start their own business. After all, we no longer work for kolkhoz but for ourselves. You just have to have a project and take out a loan. But the loans are high. It's 21% for us now. I have just taken out a loan to pay the workers. To buy chemicals and stuff.

Me: In the kolkhoz they worked with chemicals, too?

**Emilian:** Of course! And how! Hmmm… In Europe they don't like our wine.

Me: Why?

**Emilian:** Because (pause)… I can't think of the word. It is not in their assortment. Well, because I have no money, I have to do some weeding every few weeks. That is an incredible amount of work and a lot of money (…) If I had herbicides, I could put these on it. But I don't have any biological herbicides.

Me: You work the land with herbicides?

**Emilian:** No, no, no. They are way too expensive. And where am I supposed to sell the crop?

Me: So you do organic farming?

**Emilian:** That's the future.

Me: Why?

**Emilian:** How can you not understand. It's better for your health. For those who will live in this world after us.

Me: So you will do part organic and part non-organic farming?

**Emilian:** No, look. As soon as I have 100 lei together [around five Euro], I will invest them into the land. If I had more money. I would sit on the tractor and spray everything.

Me: And are there no organic herbicides or are they expensive?

**Emilian:** There is no such thing. They'd have to be imported. Here is this cap, I was at a seminar there (shows a cap with the inscription Syngenta). There they gave us this cap. This company, Syngenta, did a seminar and gave us a cap, pencil and a pad.

Me: And what did they say?

**Emilian:** That we should buy their products.

Me: Organic or not?

**Emilian:** No, no, no, no, no. For preserving agriculture I have to work with others that work ecologically (…)

Emilian and Vala are confronted with big challenges in growing and selling their grapes. Finding a feasible strategy is not easy in these changing times where farmers have to adapt over and over again to new circumstances. During the time as kolkhoz workers they were used to chemical viticulture. When they started to cultivate their fields on their own, the money for chemicals simply was not there. So they started to cultivate organic, which is very labor-intensive, with the help of their three children. Now that the children are gone, manual labor is missing. But as long as their children support them and the village is well populated, they still find workers to help care for their vines, even if it sometimes means having to borrow money to pay people. But the money is not enough for herbicides and even if they would grow more, they would not know who to sell it to, as the local market is run by the liders. So Emilian and Vala are trying to focus on buying more land so that in the future the children might be able to live on it. Because both are older and physically not able to work so much, they do not keep animals or grow enough for their own needs anymore. As long as they are financially supported by the children, they can get food from outside their farm. So they keep to their vision of building a subsistence farm that will eventually be big enough for the children to return and make a livelihood.

## Insights into Rural Topographies of Care in Moldova

This section attempts to look at crucial challenges and consequences that people cope with in rural Moldova where they find themselves at the end of agricultural care chains, while most relatives, friends and neighbors labor abroad. These chains accompany rural to rural labor mobility and migration and are structurally embedded in the agricultural sector and the care economy that has been reorganized through the international division of (re)productive labor. I aim to provide a sense of rural topographies, that is to describe the material effects of globalization on the production and reproduction of spaces and social relations and how they influence each other in rural Moldova. These topographies—that are as unique, specific and situated as they are indicative of patterns of global power relations—represent the so-called invisible economy within the Iceberg Model of Capitalist Patriarchal Economics (Mies and Bennhold-Thomsen, 1999). I will briefly focus on five interrelated patterns. This enumeration is by no means complete but rather based on the 22 testimonies such as those of Rodica, Alesja, Ina, Sergej, Emilian and Vala and on further insights from the last nine years, including studies on the Roma communities in Moldova.

### Gender Roles, Multiple Burdens and Limited Care Capacities

The interviews show that multiple burdens are put on the rural population in Moldova. Gainfully employed subsistence farmers have many spheres of responsibilities: agricultural care (considered as farm work), their additional wage job (often abroad), care activities for children or grandchildren, and further care tasks such as looking after further relatives, friends or neighbors. When people go abroad to perform wage labor, those who stay have to fill in the gap, further enhancing their burdens. Help with care work can be organized in paid or unpaid forms, but the situation is somewhat different with regard to agricultural care: When people labor abroad, it is often the next of kin that take over care responsibilities toward relatives. Women, and grandmothers in particular, are often asked to take over care for children. Sometimes people also turn to distant relatives, acquaintances or neighbors in search of support, most often following traditional gender roles. However, the rising amount of fathers and grandfathers that are taking over care responsibilities toward relatives is visible. They are increasingly taking over caring activities ascribed to women as a result of a feminization of migration patterns. Consequently, there is some movement in the gendered division of labor that is characterized by a contemporaneity of traditional gender roles along with inconsistencies as women are taking over the role of ‘breadwinners’ abroad. This is also reflected in intergenerational levels, when, for example, a daughter cannot take care of her sick mother because she works abroad and so the father takes over this role. The care of animals, plants and the soil takes place either as gainful employment or within the scope of one's own subsistence. While larger farms employ people, help in subsistence farming is hardly possible to organize. On the one hand, people cannot often afford the additional cost of paying others to help them. On the other hand, finding help in the form of mutual assistance is almost impossible in the countryside. The elderly are already subject to multiple burdens due to the absence of the middle generation, so helping others physically is hardly conceivable. This directly impacts subsistence production, as will be discussed later.

### Ethnicized and Gendered Agricultural Care Chains and Labor Relations

Subsistence farmers seldom employ workers on their farms. If they do so for specific tasks, it is often neighbors or people from neighboring villages. On the other hand, bigger farms employ day laborers. According to statistics and surveys conducted in Moldova, these day laborers often belong to the marginalized Romani people. While most former kolkhoz workers received a plot of land after the collapse of the Soviet Union and in the context of the land reform, Romani people did not. This injustice has still not been reflected upon, as Anti-Romanism remains very present in Moldova (Mihalache and Rusanovschi, 2014, 19ff) as in the rest of Europe. Compared to the rest of the population, most Romani people remain excluded from access to land due to this type of discrimination (ibid. 39). Many, predominantly women, toil as agricultural day laborers as a result of their exclusion from the labor market (ibid. 36). According to a survey with 60 Romani women, they report that their discrimination is also reflected in salaries, as has been stated in an interview: “They pay Roma 100 Lei but they pay Moldovans 120–150. But we need work, so we take the 100.” (ibid. 39). Not only do they only receive five euros per day, but their income is always uncertain. Rural Romani women often struggle to survive, performing only day labor in agriculture a few months per year (ibid. 37). Besides working on farms in Moldova, Romani women increasingly travel abroad to places like Ukraine and Russia. Agricultural labor in Ukraine or Russia takes place without a contract and under precarious conditions (Lawton et al., 2018, 11). Lack of land and an extremely precarious day labor income leads to high levels of food insecurity (ibid.). Also, it is to be expected that the agricultural (re)productive division of labor is highly gendered, as Romani women report being mainly involved in tasks such as “harvesting apples, home-based businesses” or preparing and selling food (ibid. 21). The cycles of poverty are desperate, also due to the tragic health conditions many Romani people are facing (ibid. 38) and their exclusion from education (ibid. 26). While many people in Moldova have access to the European labor market by claiming co-ethnicity and receiving Romanian or Bulgarian citizenship, this is rarely possible for Romani people. While certain agricultural care chains end in Moldova, those at the very end are the Romani people living in Moldova—those who are marginalized from access to land, wage labor, health and education. These people, and especially those who are women, toil informally not only in Moldova but also in Ukraine.

### Rural Precarity: Reduction of Subsistence, Earth Fertility and Reciprocity

Most of the time the elderly find themselves to be alone in coping with the subsistence. This is especially difficult if they additionally have to take care of small grandchildren or other community members. Usually these burdens are accompanied by a reduction in subsistence, which often includes reducing or phasing out livestock farming. Animals need unconditional and daily care that is especially challenging for older people. Villagers also, as a result, lease their fields to liders—the rural upper class. This leads to the reduction of the agricultural subsistence to a minimum. Agreements with the liders include a fixed harvest share of the leased fields. This includes the fact that in the event of a crop failure, no compensation for the lease is paid. These contractual relationships are accompanied by power relations that in no way imply that those who own land have more power. Landowners are often the ones who have to beg and fight for their harvest share from the liders. As people are facing serious limits on their ability to live off of subsistence, they additionally rely on financial support such as remittances or debts, leading to a cycle of precarity as buying food is also quite expensive. This condition also leads to an agriculture that trends toward monoculture. Rural spaces had already suffered greatly under the kolkhoz cultivation–above all, due to the introduction of tobacco cultivation, intensive livestock farming and the use of huge amounts of pesticide during soviet times. This dynamic was interrupted at some places and many fields rested for decades due to the collapse of the kolkhoz system, and the soil partly and slowly started to regenerate. However, this new development further limited reciprocity and circularity as such relations are based upon and allow for different plants to grow together at the same places that are able to create caring relations. As an overall dynamic the lider system is accompanied by further soil erosion, a reduction and destruction of Earth fertility and will lead to a decline of humus in the soil and further demise of self-regulative irritation systems.

### Vulnerable Rural Social Security Systems

Rural communities build places so that workers who are abroad can return and recover, hence allowing for a regeneration period. Also, people can find caring spaces where they are looked after if needed because they became sick or find themselves unable to continue performing wage labor abroad as a result of injury. As seasonal labor for foreign workers is not only badly paid but also without compensation for the rest of the year, people can only save some money for their future plans by coming home; there they find places where expenses are minimal because of agricultural subsistence and not having to pay rent. Rural communities hence build social security systems that people lack as wage laborers and foreign workers abroad and with limited or even no access to rights. These are not only places where the labor force for the agricultural sector and beyond is being (re)produced through childbirth and care; they also form a central back-up for the people working abroad who can return and resettle and have a place to live or to plan further steps. Those who remain therefore also carry a certain burden to obtain the agricultural subsistence and hold together the land they own by protecting it from land grabbing and expropriation.

### On Resiliency, Self-Care and Self-Defense

Owning land is by no means a guarantee of livelihood security, as lack of care capacities and challenges in maintaining the subsistence can result in quasi-possessionlessness. A further challenge is selling the food produced. However, agricultural property gives a better chance at livelihood as the most precarious, including some Romani communities, do not even have this ‘privilege’. Those who achieve a good yield by intensive care at times find themselves under pressure by local businesspeople to sell their land. Older people in particular are coming under great pressure. As the agricultural care chains end here, the physical absence of people in the countryside represents a problem due to the lack of physical, social and emotional support to cope with or to organize against the expropriation of their land. People find themselves on their own to develop self-defense strategies. Those who do not want to sell their land may also face perilous attacks. To be in the last place in the series of agricultural care chains and to maintain or even defend one's own subsistence while being thrown back on oneself and the lack of a supportive community is a quasi-impossible challenge. The tendency to sell or rent small plots leads toward a commodification of farmland and a mechanization of agricultural care that again is the basis for enterprises to come in and sell their products. Agricultural and social resiliency is hence also weakened by land grabbing of local upper classes and enterprises such as Syngenta that, in search of new investment regions, force farmers into dependencies while destroying their soil. These processes are characterized by selling hybrid seeds and poisoning the slowly recovering soil with fertilizers. Self-care and self-defense capacities are therefore generally put into question as rural spaces are put under social and agrarian existential pressure. But small-scale subsistence farming is important as a source of food security for care providing communities, most of whom have no further income. Caring for soil, animals and plants is not only a burden but comes with inter-species healing relations and circular caring practices. When these relationships fall apart, a space of self-care is also destroyed. For this reason it is important to note that when agricultural subsistence is in danger, so is self-care.

### New Dependencies: The Leader System and Remittances

Those coming from rural areas in Moldova often perform wage labor in Europe in the most precarious sectors such as in the care economy and in the agricultural and agrifood sector. If their labor relations are characterized by hypermobility, they perform wage labor only for several months a year. This situation does not allow for someone to sustain themselves, invest in the future or support whole families. Others that migrate more permanently, if not illegalized, still face the challenge that they also often labor in precarious sectors abroad that hardly allow for sustaining one’s life abroad and financially helping one’s relatives at home. While remittances are indeed helpful in particular moments, they do not and cannot compensate for the overall physical absence in rural Moldova and in subsistence production. So while remittances and the hypermobilization of workers are interwoven, both lead to a reduction of subsistence production and farmers that therefore buy more and more (increasingly imported) foodstuffs. Hence rural communities face the very difficult dynamic that they depend even more on money, transnational wage labor, remittances and credits. Whereas in the past the rural population faced inescapable dependencies within the kolkhoz/sovkhoz system, today the rural population heavily depends on the exploitative lider system and on remittances. The lider system is not meant to establish an exchange system that would allow for food sovereignty of subsistence farmers; instead, it puts people into new dependencies, as relying on remittances represents a transnational and neocolonial dependency infrastructure. Both liders and remittances cannot and do not aim for a sustainable solving of the fundamental, local problems. In fact, discourses around remittances and triple-win solutions within the rising remittance infrastructure represent colonizing discourses with the hegemonic effect of linking the understanding of wealth and well-being to money flows and GDP.

## Fragments of the Permanent Subsistence Crisis and Landscapes of Wounds

Studies on the global care economy, care chains and transnational caring relations have highlighted new possibilities of care from a distance through virtual communication. The testimonies above (see Rodica, Alesja, Ina, Sergej, Emilian and Vala) show how agricultural care chains cannot be compensated for at the end of these chains. Caring from a distance is not an option here. Rather, caring within subsistence production relies on continuous physical presence and encompasses caring for, about, with and through the surrounding landscapes. I argue that agricultural care chains within rural to rural migration and labor mobility regimes, among other factors, negatively impact well-being in rural Moldova. I further argue that it even hinders an exodus out of rural precarity, as it is a precarity deeply inscribed into landscapes and social relations for many generations and that is therefore especially hard to cope with.

In order to understand this rural precarity as a whole—and as a basis to look at well-being in rural Moldova—it is necessary to contextualize the everyday struggles we have seen above, linking them to the experiences of their communities and to the past by tracing back deeply rooted and omnipresent wounds in the villages. When talking about present challenges, people still refer to the aftermath of forced top-down collectivization during Soviet times and explicitly contextualize their life course within the later post-Soviet processes of decollectivization (see Emilian and Alesja). Those who have lived in rural Moldova for generations often remember and talk about how their relatives experienced land expropriation or how they fought to receive land in the decollectivization period, while confronting a variety of struggles that accompanied these big changes in their communities. The complexity of this time, including experiences of violence and peasant resistance cannot be even rudimentarily done justice here. I argue that the wounds of these experiences are represented today in rural Moldova through transgenerational and environmental trauma that has not been worked through or even recognized. What trauma am I referring to and what do I mean by this? Following the reflections on decolonizing trauma studies (Linklater, 2011; Andermahr, 2016), I understand trauma in this context as shared experiences of danger to life and of subordination as well as experiences of forced expropriation of land that continuously harmed and shaped whole communities over generations. These experiences are inscribed in the individuals just as they are inscribed in the relations between them and others and into the landscapes. What does this mean in the case of rural Moldova?

In order to understand and reflect on recent wounds we have to look into the historical traumas (Brave Heart-Jordan, 1995) of this region: Under the leadership of Stalin, beginning in 1929, private farms were merged into so-called collective farms. With this forced collectivization in the period of the first five-year plan, Stalin ushered in the strongly centralized planned economy era. This violent collectivization aimed for the "liquidation of the kulak class" (Stalin). The so-called kulaks^5^ (wealthy farmers) were expropriated without replacement and deported to ‘uninhabited’ areas or killed (Altrichter and Haumann, 1987; Baberowski, 1998). Poor peasants, on the other hand, became workers on the kolkhoz/sovkhoz farms. Consequently, every family shares memories and painful experiences of these times. These processes were accompanied by rural exodus in Moldova such as in other regions of the Soviet Union, as the rural population faced mass famine. In order to control the urban and the rural population in the 1930s and to feed growing cities and industrialization in the name of productivity growth, soviet citizens were given passports. Peasants were excluded from this practice, however, and tied to the land by being forced into food production (Данилов et al., 2001). In the name of ‘liberation’, the poor rural population again faced living conditions that were similar to serfdom experiences of previous centuries. As the kolkhoz/sovkhoz system was one of exploitation and subordination, workers had almost no say in the means of production or free choice on labor relations and were bound to live as poor, rural workers. Only in the 60s were peasants granted passports (Engebretson, 2007, 13). Today, people that identify themselves and their families as Moldavians describe how they were suddenly faced with ‘people from Moscow’ sent to tell them what to do. Others remember how they were expected to learn and to speak Russian and how their families experienced a devaluation of their communities (life stories from further interviews that are not included here). I argue that ethnicized conflicts in Moldova were and remain experiences that are to be analyzed within the decolonial trauma studies framework of ethnostress (Antone and Hill, 1992). Soviet policies of indigenization (see κоренизация/korenizatsiya) had complex and occasionally contradictory effects on ‘ethnic’ inclusion and exclusion (King, 1998). All of the rural population in fact suffered, but the forced collectivization and implementation of the kolkhoz/sovkhoz system affected people differently. This is visible when listening to their stories and is a viewpoint also shared by Igor Caşu who analyzed the social and ethnic composition of victims from that time. He therefore suggests that “Stalinist terror in Soviet Moldavia should not be categorized as ethnocide, but rather as genocide or a crime against humanity” (Caşu, 2010, 53). While it has been recognized in settler societies, postcolonial regions and in regard to religious or ethnicized/racialized minorities—to a certain degree at least—that such experiences have long standing effects on communities, this has not been reflected on in relation to Moldova.

Not only did the forced collectivization during Soviet rule establish long standing toxic labor relations and living conditions regarding social and health aspects for the rural workers, it also accelerated environmental destruction and inscribed itself into the landscapes as wounds. Atomic gardening was introduced (Turea, 2019) and Khrushchev together with the Communist Party decided that Moldova should become the “orchard of the Soviet Union” (Low, 1963, 14). Moldova was constructed and remains romanticized as the ‘fruit garden’ and ‘winery’ of the Soviet Union. The cultivation of slops and the desiccation of water-sides and swamps led to the acceleration of water and wind erosion for soil on slopes and the salinization of soils in the watersides (Summer and Diernhofer, 2003, 25ff). This—together with the mechanization of agricultural production, the introduction of tobacco plantations (see the experiences of Aljesa), the intensive use of fertilizers and pesticides and an increase in livestock farming—resulted in adverse conditions for flora and fauna in general and in an agricultural system that over centuries led to the distraction of soil and earth fertility. It is important to mention that such environmental destruction did not begin with Stalin’s collectivization, and hence did not occur as an hour-zero-dynamic but was built on the already intensive deforestation in this region beginning in the 14th century as a result of colonization and wars. The exploitation of forests in this region especially accelerated in the 17th to 20th centuries under the rule of the Ottoman and Russian empires (Cocîrţă, 2012, 61). “Compared with 1812 forest ecosystems in the Dniester-Prut area decreased from 450,000 to 160,300 ha in 1914: practically been eliminated over two thirds of the forested area.” (Cocîrţă, 2012, 64). Decades before and during Soviet rule, deforestation was already a recognized problem as it caused soil degradation and soil erosion and led to afforestation programs (Chendev et al., 2015). Though some rise in forestry in Moldova occurred (Cocîrţă, 2012, 63), the programs have not been successful on a bigger scale (Brain, 2010). Today, compared to the surrounding countries, Moldova has by far the smallest proportion of forest area (about 12,5%) in relation to the country's surface area (World bank, 2020).

These *ecological wounds* represent themselves on the Earth's surface as landslides and as gullies that have been formed slowly over decades—not at least due to intense deforestation of the previous centuries under colonial rule. Trees are a central component of a functioning ecosystem that allow for reciprocity and circularity between different species, leading to humus formation. Trees also tame the wind, attract water and strengthen the resilience of the soil and its fertility. If trees are missing, then protection and care (of the soil) is missing, too. On top of this, the division of people into rural workers and engineers on farms and the expropriation of land not only harmed people but also accelerated the ongoing destruction of local knowledge of living in and with ‘nature’. After the collapse of the Soviet Union and within the context of “ethno-national mobilization” (Crowther, 1991), new traumas emerged (Abbott, 2007). While social wounds were deepened, the very same happened with the already tormented soil. The privatization of the land due to decollectivization namely had further negative impacts on the soil and Moldova risks losing its most valuable resource—the fertility of the chernozem (Summer and Diernhofer, 2003, 26), that is, the black soil that is rated among the most fertile because of its high percentage of humus.

While gullies based on environmental and ecological traumas that need healing are very visible as open wounds in the landscapes in rural Moldova, intergenerational traumas are harder to grasp, hard to name, and yet, I argue, they weigh heavily in social relations. The effects of trauma on communities in Moldova that have over many generations suffered under changing repressive political regimes and environmental extraction need serious engagement. I can only hint at a few aspects that are visible at the edge of the testimonies presented and omnipresent in society: alcoholism and violence against women in intimate relations (see Lena, Rodica’s sister), attacks on and threats to the livelihood of poor subsistence farmers (see Aljesa) and underdeveloped solidarity structures in villages along with a lack of collective care practices and trusting communities. Alcoholism and domestic violence have already widely been recognized as problems. Moldova has the highest alcohol consumption in the world, especially in rural regions and men are especially affected (World Health Organization, 2018). While alcoholism is related to morbidity and mortality, it is furthermore the strongest risk factor associated with domestic violence in Moldova (Ismayilova, 2015). A study shows that seven out of ten women from rural areas of the country (six out of ten in urban areas) have suffered at least once in their lifetime from violence in intimate relations (World Health Organization et al., 2016). Widespread alcoholism, in the same way as violent relations in communities that experienced colonialism and ongoing structural discrimination, has already been recognized as a symptom of historical and transgenerational trauma (Antone and Hill, 1992; Brave Heart-Jordan, 1995). Therefore questions of healing have become an important community-based approach among those that experienced “massive losses of lives, land, and culture from European contact and colonization resulting in a long legacy of chronic trauma and unresolved grief across generations” (Brave Heart and DeBruyn, 1998). Although the trauma of neocolonialism has also been put on the table (Švarc, 2013), neither Moldova’s past colonial power relations nor recent neocolonial experiences of subordination, exploitation and discrimination within or outside the country have so far been situated within this context, nor have the ongoing experiences of intergenerational (re)traumatization been analyzed regarding their effect on individuals and communities in order to approach healing perspectives.

Whereas in general community-level effects of trauma are indeed perhaps the most insidious and the least studied and understood (Evans-Campbell, 2008, 327), decolonial trauma approaches are urgently needed in the case of Moldova and represent an important area for further investigation. I want to briefly point to a crucial symptom that I mentioned above. This symptom is represented in all testimonies and is to be considered in the analysis of post-Soviet community trauma in rural Moldova. It also serves as a point of departure for theorizing agricultural care chains: The underdeveloped solidarity structures in villages and lack of collective care practices and trust in communities. We have seen how people are thrown back on themselves in their everyday lives. It is not only Rodica, Alesja, Ina, Sergej, Emilian and Vala who are relatively isolated and alone when dealing with structural challenges and care needs on their farms. Apart from global power relations, subsistence farmers also face local power relations and at times even attacks (see Aljesa) that hardly allow for well-being and living on subsistence production (especially see Ina and Sergej). Furthermore, people do not trust each other in general or in everyday life and do not aim to organize agricultural production together with others but are instead thrown back to the liders (see Ina and Sergej). I argue that when it comes to building alternative agrarian structures that would allow for well-being in rural Moldova, people do not only face power relations they can hardly handle and that hinder such movements and critical endeavors, but they also carry historical traumas that have not been addressed so far. Pinderhughes and colleagues argue that one of the symptoms of community trauma is “a low sense of collective political and social efficacy” along with further symptoms such as intergenerational poverty, limited employment, long-term unemployment and deteriorated environments (Pinderhughes et al., 2015, 13)—all factors that are represented in (rural) Moldova. The low sense of collective political and social efficacy must be given special consideration against the background of post-Soviet rural experiences of collectivization and the kolkhoz/sovkhoz farms. The farming system that has been declared to be ‘collective’ (kolkhoz is a shortcut of KOллeκтϰBHOE xoзяncTBO/kollektivnoye khozaystvo, meaning collective economy), I argue had a major impact on individuals, social relations, communities and the imaginations of people in post-Soviet regions. Beyond creating individual and transgenerational trauma due to the violent experiences of past decades, it affected the everyday life of communities and is inscribed into the structures of villages. The term collective and even more so, collective as a concept is closely tied to the darker side of history (apart from nostalgic people, who also exist), and is hence linked to experiences of subordination, compulsion and a failed system. Collective in the cultural archive (Said, 1993) has been linked to a state-controlled authoritarian organizing principle. It is for this reason that there is barely even a discursive space for addressing alternative collective organizing.

This might be one of the biggest traumas—that the Soviet Union and the politics of forced collectivization and its aftermath have inflicted on people and whole communities: It occupied imaginations of collectivity and collective power. Instead of being a healing process, decollectivization fragmented farms and separated people while capitalist entrepreneurship (see the lider system but also the interview with Emilian) developed as the only post-socialist way of organizing (agriculture). Lone fighters become the dominant mode of existence. Socially, this meant that everyone was fighting for survival according to the strategy that everyone secures what they can for themselves. Luckily, practices and worldviews that contradict these dynamics exist, most visibly in every day caring relations, as we have seen in the testimonies. They represent seeds toward healing practices but also toward decolonizing agricultural care chains. They show how people refuse the integration of their agricultural production into the international food market, develop relations of mutual aid and solidarity and do reciprocal care within relations of living in and with ‘nature’ (see Aljesa). They furthermore consider further generations and refuse to use toxic fertilizers to poison the soil (see Emilian), do not exploit others for their own reproduction but handle what they can and are capable of (see Rodica) and still believe in the idea of organizing things together (see Ina). Finally, and in the context of a personal endeavor, people work through their Soviet and post-Soviet traumas while developing healing practices and strategies of self-determination (see Aljesa).

I argue that following agricultural care chains to their ends and looking at the effects of these phenomena on subsistence production in Moldova reveals contours of long-standing rural precarity that manifest as a permanent subsistence crisis. Following Mies, I understand subsistence production as “all work that is spent in the production and maintenance of life and also has this purpose” (Mies and Bennhold-Thomsen, 1999), but the understanding of what constitutes a crisis needs to be sharpened. Whereas the global care crisis (Isaksen et al., 2008) has also been referred to as “permanent reproductive crisis” (Federici, 2013), the hegemonic framing of crisis within the subsistence crisis discourse is rather narrow. Subsistence crisis in the European context has so far been understood as almost tantamount to a famine or hunger crisis, though as a crisis “of lesser intensity” (Bass, 2010, 141). Within this context the so-called European subsistence crisis of 1845–1850 has been referred to as “the last European subsistence crisis” (Gráda et al., 2007). It has been considered a state of emergency for a limited time that is mainly characterized by crop failures, increase in prices of basic foodstuffs, decline in real wages, lack of food and accompanied by a starving population and hunger-induced illness as well as high rates of mortality, mobility and low rates of population fertility, marriage and economic growth.

I propose a different understanding of the subsistence crisis, one that is able to grasp the recent precarity in rural Moldova and other regions. Understanding of this crisis can not depart from a definition as a rather isolated and self-contained problem of the past; rather, the broader conditions of the “Iceberg Model of Capitalist Patriarchal Economics” (Mies and Bennhold-Thomsen, 1999) must be taken into consideration. Following the testimonies above and the idea of current multiple crisis (Vielfachkrise according to Demirović and Maihofer, 2013), I challenge this hegemonic understanding of a subsistence crisis. Demirović and Maihofer propose an understanding of crisis that attempts to take into account the diversity and inherent logic of various crisis processes. They assume that crises are forms of autonomous (protracted or rapidly destructive) processes, each of which has a specific character resulting from social conditions and concrete conflicts (ibid. 32). Which social processes and phenomena are determined to be crises differs for different persons or groups while the central mechanism of domination is to not allow the crisis dynamics to be grasped in their internal context—to isolate or shift them socially, spatially or temporally (ibid. 33). According to Demirović and Maihofer, crises are always crises of concrete contexts of domination and therefore crisis dynamics and crisis phenomena always form an internally interconnected context (ibid. 34).

Following insights into rural Moldova and these elaborations, I redefine the subsistence crisis as a permanent yet contested and changing state of ‘rural’ precarity and vulnerability (see Butler, 2012, especially 141ff.) that has existed since primitive accumulation and enclosure (expropriation of the commons)—in Europe (see Federici, 2004) and in the colonies as well as in settler societies-that expresses itself differently depending on time and space. Within this crisis, life and subsistence production is put under threat, while contours and possibilities for change are exposed. The subsistence crisis is mainly characterized by the radical devaluation of ‘nature’ and subsistence production in general and the appropriation of care, care capacities and caring spaces for potential short-term productivity growth and capital accumulation. This crisis has to be understood as a global crisis that tends toward the sustainable destruction (following “sustainable underdevelopment” by Spivak, 2012) of reciprocity, circularity and resilience in rural spaces. Due to the colonial history and contemporary coloniality (Quijano, 2000; Lugones, 2007; Maldonado-Torres, 2007; Tlostanova, 2012), the permanent subsistence crisis involves the worldwide destruction of knowledge and ways of living in and with ‘nature’ such as the gendered racialization/ethnicization of people along with a dehumanization of ‘the other’ that is seen as being closer to ‘nature’ and as a ‘free resource’ to be exploited. Following Cindi Katz, I argue that identifying contour lines and mapping countertopographies enables connecting places beyond Moldova that are differently affected by this subsistence crises.

## Conclusion and Outlook–Decolonizing Well-Being, Collectivity and Subsistence Production

By mapping the very concrete conditions of agricultural care chains, rural precarity not only becomes visible but leads me to contextualize this precarity within a broader understanding of a global and permanent subsistence crisis. This crisis–though it can be considered as an autonomous crisis with its own dynamics and specifies–is strongly interconnected to other crizes and therefore manifolds very localized contours of a multiple crisis (Demirović and Maihofer, 2013), further perpetuating exploitation and intersectional marginalization according to age, ethnicity, gender, class and neocolonial experiences. We have seen how in the context of Moldova the subsistence crisis is linked to the recurrent destruction and expropriation of collectivity and collective labor as well as to the environmental crisis, the crisis of masculinity (Zdravomyslova and Temkina, 2013) and the gendered health crisis (Field, 1995; Hinote and Webber, 2012).

Diversifying the single story about Moldova as the “country without parents” and reframing it as one of the regions that carries the world on its shoulders could be a decolonizing narrative. This can only be understood by taking into consideration the structural conditions of agricultural care chains. These chains that hinder an exodus out of rural precarity not only make visible how rural Moldova forms a part of the invisible economy (see the Iceberg model) of today's neocolonial food production regimes in Europe, they also show how wealth and poverty are fundamentally interconnected according to the international division of (re)productive labor within the neocolonial agricultural labor regimes in Europe. So while following Mies and Bennhold-Thomsen (2000, 5), I agree that—among other liberating steps—various kinds of local and transnational oppressors need to get off the backs of rural communities in Moldova and additionally that Romani people need access to land. A transnational perspective on the abolition of the international division of (re)productive labor is also inevitable.

In the context of Moldova, this division comes on top of post-Soviet traumas (Wakamiya, 2011) and needs special attention in terms of identifying localized perspectives and challenges to healing. At the same time, following the contour lines of the subsistence crisis and looking into further topographies of formerly colonized regions, it can be seen that countertopographies build the basis of developing common struggles and allow for an exchange of urgently needed healing practices. Such an endeavor is urgently needed and inevitably linked to further local politics of underdeveloped solidarity structures in villages that find themselves on the other end of agricultural care chains as many European countries withdraw from farming. Villages in wealthier regions in many European countries that have increasingly been converted into bedrooms or holiday resorts for the middle and upper classes also face a lack of collective care practices in organizing agricultural production together. Therefore, partially due to their own financial challenges, farmers periodically recruit workers from peripheral regions and send them back when not needed. Hence, a liberating perspective would include decolonizing collectivity in the context of post-Soviet/postcolonial experiences and decolonizing subsistence production in wealthier regions in the context of “learning to unlearn” (Tlostanova and Mignolo, 2012) what counts as progress and what constitutes well-being. This would include decolonizing our mindsets and social relations (Walia, 2013, 250ff) as well as developing different access to subsistence production and building “caring communities” (see Sorgegemeinschaften, Precarias, 2011, 104ff) in rural spaces such as independent and decentralized bridges toward cities that could on an ethical and material level link the well-being of every village to the well-being of every other village.

The subsistence crisis reveals landscapes of deep wounds in Moldova–wounds that are based on centuries of subordination under different empires and oppressors. It has led to individual and community trauma, the sustainable destruction of circularity, reciprocity, the soil and knowledge transfer about living in and with ‘nature’. This article is limited because a deep engagement with traumas in Moldova is not within the scope of my research. Instead, it serves as a first step in opposition to different single stories as well as to empirical, epistemological and disciplinary narrowings; it is a proposition for thinking about the nexus of caring relations and the subsistence crisis that is informed by trauma studies and that is aiming for research toward healing practices within a decolonial, abolitionist perspective.

## Data Availability

The interviews supporting the conclusions of this article are not readily available because they are confidential as agreed upon with the participants. Questions should be directed to Dina Bolokan, bolokan@protonmail.com.

## Footnotes

1 While I mostly refer to Moldova, it should be pointed out here that Transnistria is neither excluded nor analyzed separately within this article.

2 As a reminder: Marx assumes that the ‘success’ (that is capital accumulation) of capitalism lies in the fact that it can use a resource, namely the *human labor force,* without having to spend as much value on it as it produces. This human labor force has been equated in theory and political practice to the heterosexual, white male worker in the factory.

3 In turn, Rose Luxemburg (1913) in her theory of imperialism assumed that capitalism must always be imperialist in order to access new supply sources; that is, it must always incorporate non-capitalist areas and elements, and at that time, of course, this meant especially nature and unpaid work in and from the colonies.

4 I define the elder population as the age around retirement; the retirement age in Moldova for women is 57/58 years and 62/63 for men.

5 Kulak means fist in Russian. The term was used to describe wealthy farmers. Also see dekulakization (Russian: раскулачивание).
