# Erratum: Against Single Stories of ‘Left Behind’ and ‘Triple Win’: On Agricultural Care Chains and the Permanent Subsistence Crisis

**DOI:** 10.3389/fsoc.2021.723390

**Published:** 2021-06-17

**Authors:** Frontiers Production Office

**Affiliations:** Frontiers Media SA, Lausanne, Switzerland

**Keywords:** labor migration, international division of reproductive labor, agricultural care chains, Moldova, well-being, subsistence crisis, decolonization, post-soviet studies

Due to a production error, an incorrect version of the article was published. The correct version has now been published and the earlier published version has been included as Supplementary Material to preserve a record.

The publisher apologizes for this mistake. The original article has been updated.

